# COVID‐19 therapy with mesenchymal stromal cells (MSC) and convalescent plasma must consider exosome involvement: Do the exosomes in convalescent plasma antagonize the weak immune antibodies?

**DOI:** 10.1002/jev2.12004

**Published:** 2020-11-14

**Authors:** Philip W. Askenase

**Affiliations:** ^1^ Section of Rheumatology and Clinical Immunology Department of Internal Medicine Yale University School of Medicine New Haven Connecticut USA

**Keywords:** cell therapy, COVID‐19 convalescent exosomes, convalescent plasma, COVID‐19 antibodies, COVID‐19, exosomes, extracellular vesicles, mesenchymal stromal cells, miRNA, nanoparticles

## Abstract

Exosome extracellular vesicles as biologic therapy for COVID‐19 are discussed for two areas. The first involves the growing use of mesenchymal stromal cells (MSCs) for the profound clinical cytokine storm and severe pneumonia in COVID‐19 patients. Instead, it is recommended to treat alternatively with their MSC‐released exosomes. This is because many reports in the literature and our data have shown that the release of exosomes from the in vivo administered MSC is actually responsible for their beneficial effects. Further, the exosomes are superior, simpler and clinically more convenient compared to their parental MSC. Additionally, in the context of COVID‐19, the known tendency of MSC to intravascularly aggregate causing lung dysfunction might synergize with the pneumonia aspects, and the tendency of MSC peripheral vascular micro aggregates might synergize with the vascular clots of the COVID‐19 disease process, causing significant central or peripheral vascular insufficiency. The second exosome therapeutic area for severe COVID‐19 involves use of convalescent plasma for its content of acquired immune antibodies that must consider the role in this therapy of contained nearly trillions of exosomes. Many of these derive from activated immune modulating cells and likely can function to transfer miRNAs that acting epigenetically to also influence the convalescent plasma recipient response to the virus. There is sufficient evidence, like recovery of patients with antibody deficiencies, to postulate that the antibodies actually have little effect and that immune resistance is principally due to T cell mechanisms. Further, COVID‐19 convalescent plasma has remarkably weak beneficial effects if compared to what was expected from many prior studies. This may be due to the dysfunctional immune response to the infection and resulting weak Ab that may be impaired further by antagonistic exosomes in the convalescent plasma. At the least, pre selection of plasma for the best antibodies and relevant exosomes would produce the most optimum therapy for very severely affected COVID‐19 patients.

## 1 COVID 19 THERAPY WITH MESENCHYMAL STROMAL CELLS

### 1.1 Introduction to the wide clinical usefulness of MSCs

MSCs have been safely administered to humans in many clinical instances over the past 25 years. Commonly employed harvest sites for in vitro expansion of MSC precursors include bone marrow, adipose tissue, placenta and umbilical cord. These antecedents of the employed MSCs are present in these diverse connective tissue sites at a very low concentration; in the range of one per 10,000 cells (0.001%) (Saeedi, Halabian, & Imani Fooladi, 2019).

Since MSCs are self‐replenishing, adherent to culture apparatus surfaces and do not have specific surface markers of most immune and myeloid cells, they can be grown in vitro to huge numbers; up to 100% for use in vivo. Culture is performed on increasingly large surface areas of sterile flasks under strict aseptic conditions. The phenotype of MSC and MSC‐derived exosomes (MSC^exos^) is positive for major histocompatibility Class (MHC) I, CD73, CD90 and CD105, and their exosomes also express typical surface marker tetraspanins (CD9, CD63 and CD81). They do not express typical T cell, B cell, macrophage, myeloid or embryonic cell surface marker antigens, and generally also no expression of MHC‐II (HLA DR in humans) nor positive costimulatory surface molecules like include CD40, CD40L (Saeedi et al., 2019). Overall, this suggests immunomodulatory rather than effector properties. In this communication, the term exosome is used for the subset of endosomal multivesicular body‐derived small extracellular vesicles (sEV) of 50–150 nm, pelleting at 100,000 g, with a certain buoyant density and expression of surface tetraspanins and other markers, and are capable of transferring their contents to other targeted cells; including miRNAs to induce epigenetic alterations resulting in important functional changes (Jeppesen et al., 2019).

Administering MSC intravenously (IV) to rodents with induced models of clinical diseases at just a million cells, can result in reversal of abnormalities for weeks thereafter. All told, these effects are nutritive, trophic, reparative, anti‐inflammatory, and anti‐immunologic; as well as healing of abnormalities in the micro vasculature (Hoffman & Dow, 2016). Thus, regarding very ill patients with COVID‐19 infections, MSC may be ideal for decreasing the pathogenesis of severe viral pneumonia and resulting acute respiratory distress syndrome (ARDS). In addition to infection, there are two accompanying potentially severe and life‐endangering immunologic syndromes that MSC^exos^ may have special properties to mediate beneficial treatment. The first is cytokine storm induced by the over reacting immune system, occurring in severe viral diseases (Huang et al., 2005; Mehta et al., 2020). The second is emerging Kawasaki‐like Multisystem Inflammatory Syndrome (MIS) of Children also resembling toxic shock syndrome (Belhadjer et al., 2020; Bilaloglu et al., 2020; Riphagen, Gomez, Gonzalez‐Martinez, Wilkinson, & Theocharis, 2020; Schroeder, Wilson, & Ralston, 2020; Verdoni et al., 2020).

### 1.2 Likely immune mediated paediatric MIS as a sequela of COVID‐19 infection that potentially could be benefitted by MSC therapy

MIS in children is a sequela of COVID‐19 that can occur after seeming recovery as a late emerging different delayed immune response. Clinically there is unrelenting fever, skin rash and some of the following: non‐purulent conjunctivitis or muco‐cutaneous inflammatory oedema (oral, hands or feet), generalized extremity pain and gastrointestinal problems like diarrhoea, vomiting or abdominal pain. Systemically, there can be hypotension progressing to shock, that is refractory to volume therapy and eventually requiring inotropic support. It is due to a dominant myocarditis with severe inflammation causing left ventricular dysfunction, pericarditis, valvulitis or coronary inflammation with aneurisms; eventuating progressively in some patients to acute heart failure or cardiac arrest (Belhadjer et al., 2020; Bilaloglu et al., 2020; Mehta et al., 2020; Riphagen et al., 2020; Schroeder et al., 2020). Similar to toxic shock syndrome and cytokine storm there appears to be a diffuse systemic inflammatory process with development of small pleural, pericardial, and ascitic effusions (Belhadjer et al., 2020; Riphagen et al., 2020). Laboratory tests show elevated Troponin and cardiac enzymes, as well as evidence of coagulopathy (by PT, PTT, and elevated d‐dimers) (Varga et al., 2020) with elevated markers of inflammation (ESR, C‐reactive protein or procalcitonin), without any microbial infectious cause. Treatment with intravenous immune globulin (IVIG), the previous approach to perhaps related Kawasaki syndrome, can be associated with recovery of left ventricular systolic function. Therapy with MSC has not previously been contemplated and might be considered in only the most extreme cases as a scientifically unexplored desperate measure.

### 1.3 Cytokine storm and pneumonia in severely ill patients infected with COVID‐19 virus

The cytokine storm occurs during primary active COVID‐19 infection. It is due to rapid release of a variety of inflammatory cytokines from viral‐activated innate and then acquired immune cells that are overreacting to the initial acute persistent viral infection (Mcgonagle, Sharif, O'regan, & Bridgewood, 2020). Clinically, there are high fevers, oedema, great muscle aches, extreme fatigue and nausea with multiple organ damage. There also is rapid developing patchy bilobar pneumonia with pulmonary oedema and dysfunctional air‐exchange. This is indicated by dangerously low pO_2_; all often progressing to ARDS, i.e. life‐threatening lung injury that allows fluid to leak into the lungs that is induced by direct viral infection of alveolar cells. The pneumonia is visible as inflammatory lesions with ground‐glass opacity on CT scan. Along with acute cardiac injury, the heart–lung injury can in some lead to death (Mehta et al., 2020). Considerable vascular abnormalities are due to vessel injury and inflammation induced by direct infection of the endothelium (Varga et al., 2020).

This severe COVID‐19 cytokine storm syndrome is primarily a virally triggered macrophage activation syndrome (Zhang et al., 2020). Accordingly, there is preferential production of a variety of macrophage activating soluble factors such as: IFN‐γ, its associated IP10 chemokine, monocyte chemoattractant protein 1 (MCP1) and monocyte chemoattractant protein 1 (MIP10); together with granulocyte‐colony stimulating factor (GSCF), as well as IL‐2, IL‐6, IL‐7 and TNF‐α  (Magro, 2020, Matsushita et al., 2015). The rises in macrophage released and directed cytokines are accompanied by severe lymphopenia; especially reduced CD4+ and CD8+ T cell counts. The severe CD3+ T cell depletion with bursts of IL‐6 and IL‐8 cytokines together are particularly bad prognostic signs, resulting in a clinical therapeutic focus on agents that interfere with IL‐6 (Magro, 2020; Mcgonagle et al., 2020; Rahmati1, 2020; Zhang et al., 2020).

### 1.4 Numerous observations that MSC used for healing therapy are not found at their apparent site of action

A crucial point is that when labelled MSC are given they seldom are found in the organ systems they are repairing. Numerous studies prove this is because they are primarily trapped in the lungs, where they are eliminated by 24–48 h after administration (Matsushita et al., 2015); during which they release their MSC‐derived exosomes (MSC^exos^) that actually are responsible for the healing processes that the MSC mediate. As noted, MSC can be harvested from precursors in a variety of connective tissues and greatly enriched by simple cultures for use as trophic and anti‐inflammatory natural healing cells. Over the last several years, it has been shown repeatedly in many systems that there is no evidence that MSC go to their targeted organs that are prospering from this treatment (Yin, Wang, & Zhao, 2019).

### 1.5 It is now established, that the healing, trophic and anti‐immune and anti‐inflammatory actions of administered MSC are due to their released exosomes

Indeed, the therapeutic effects of systemic MSC can be replicated by systemic transfer of exosomes produced and secreted by the MSCs (MSC^exos^) in a variety of experimental injury models like myocardial infarction (Sasse et al., 2020; Tan, Floriano, Nicastro, Emanueli, & Catapano, 2020), with arrhythmia (Sadraddin et al., 2019). Similarly in: CNS ischemic stroke (Xin, Li, & Chopp, 2014; Zhang, Buller, & Chopp, 2019), traumatic brain injury (Xiong, Mahmood, & Chopp, 2017; Yang et al., 2017), spinal cord injury (SCI) (Lankford et al., 2018), ischemia‐reperfusion renal injury and dysfunction (Li, et al., 2019; Tsuji, Kitamura, & Wada, 2020), liver drug injury, ischemia and fibrosis (Lou, Chen, Zheng, & Liu, 2017; Nong et al., 2016), lethal irradiation enteropathy (Chang, Qu, Wang, & Dong, 2015), and hematopoiesis restoration (Yang, Balakrishnan, Torok‐Storb, & Pillai, 2012), bronchopulmonary dysplasia (Willis et al., 2018) and bleomycin pulmonary fibrosis (Elliot et al., 2017; Mansouri et al., 2019).

In these various preclinical experimental disease models, few, if any, of the systemically administered MSCs were detected at lesion sites that were healing and further, their derived exosomes were found to deliver almost identical healing.

Additionally, MSC^exos^ replacing their parental MSC modulate immune functions (Baquir & Hancock, 2017; Chen et al., 2016), and consequently there is similar data as the above in models of autoimmune diseases; such as: systemic lupus (Perez‐Hernandez, Redon, & Cortes, 2017; Sharma et al., 2017; Zhou et al., 2020), the collagen sensitivity model of rheumatoid arthritis (Chen, Wang, Xia, Yan, & Lu, 2018; Li, Li, Liu, Gao, & Li, 2019). Type I diabetes mellitus (Chang & Wang, 2019; Nojehdehi et al., 2018) inflammatory bowel disease (Mao et al., 2018), and the experimental autoimmune encephalomyelitis model of multiple sclerosis (Farinazzo et al., 2018; Jafarinia, Alsahebfosoul, Salehi, & Eskandari, 2020; Riazifar et al., 2019).

Finally, there are beginning studies of rodent models of MSC^exos^ therapy in Alzheimer's disease (Reza‐Zaldivar et al., 2018), Parkinson's disease (Jarmalavičiūtė, Tunaitis, Pivoraitė, Venalis, & Pivoriūnas, 2015) status elepticus (Long et al., 2017) and even autism (Alessio et al., 2020). Al together this is a prodigious list. Therefore, it can be seen clearly that so‐called ‘cell therapy’ with MSC is rightly giving way to administration of their derived MSC^exos^. Further, it is also readily apparent that a very wide number of clinical tissue and organ injury diseases, as well as a variety of inflammatory, fibrotic and degenerative diseases have been found potentially susceptible to the unusual and wide healing properties of MSC^exos^ instead of treatment with their much more complicated parental MSC. It is our contention that this also applies to the use MSC^exos^ rather than the MSC in the treatment of patients with severe COVID‐19 clinical syndromes.

### 1.6 Results of MSC treatment of spinal cord injury (SCI) show that MSC^exos^ mediate the tissue repair and functional recovery

We confirmed the failure to find IV delivered MSCs at their apparent site of action in our studies of MSC‐induced healing of SCI in rats. IV injected fluorescent‐labelled MSCs were not detected at lesion sites in the spinal cord, that nonetheless improved (Lankford et al., 2018). IV infused green fluorescent protein (GFP)‐expressing MSCs trafficked transiently to the lungs, spleen and liver, where they survived for only a few days (Matsushita et al., 2015), and none were detected within the contused spinal cord that was healing (Lankford et al., 2018).

This argued that the therapeutic effects are not mediated by MSC local replacement of damaged cells at the SCI site, nor by MSC paracrine effects at the site nor unlikely long distant SCI‐directed effects on local tissue repair at the site of injury. Instead, it indicates that the exosomes alone secreted by the MSC acted in an endocrine manner while stationed in SCI‐distant organs like the lung and lymphoid organs. There, over a couple of days the MSC release MSC^exos^ into the general circulation, to then target and affect the distant damaged SCI tissues, to actually be responsible for the SCI healing and trophic effects (Lankford et al., 2018). The proof was that the in vitro culture‐derived MSC^exos^ administered IV mediated all of the in vivo actions of the MSC and when the exosome EV were labelled they could be localized as preferentially targeting a subset of healing M2‐type macrophages at the site of SCI (Lankford et al., 2018).

### 1.7 The value of clinical therapy with MSC and MSC^exos^ in patients with a variety of diseases

The above discussion dealt with properties of MSC and MSC^exos^ in a large variety of disease models in experimental animals; a necessary prerequisite to human use. The natural initial current dominance of clinical studies employing MSC is reflected in the ten human disease trials listed at the NIH clinical trials.com site that include: diabetic nephropathy, multiple sclerosis, non‐healing wounds and burns, lupus nephritis, allogenic transplantation, acute renal injury, refractory thrombocytopenia and liver failure. The more pertinent and preferential use of MSC^exos^ instead of MSC, that we are recommending here, is considered more appropriate for the exigencies and diverse clinical environmental sites of hospitalizations involved with very ill patients suffering from severe clinical COVID‐19 syndromes.

### 1.8 Therapy with MSC^exos^ in patients with relevant pulmonary diseases

At the NIH clinical trials.com web site there are listed ten projects using MSC^exos^ therapy; most in various cancers, but two relevant here. One for bronchopulmonary dysplasia (Kourembanas, 2020) and another very interestingly testing MSC^exos^ as a respiratory aerosol treatment in normal volunteers and patients with COVID‐19 pneumonia. This is not possible with cells like the MSC. This study follows on phase I/II trials with human bone marrow‐derived MSCs found to be safe for administration to patients with ARDS and septic shock (Abraham & Krasnodembskaya, 2020), and preclinical studies of therapeutic use of MSC^exos^ in acute lung injury (Lee, Park, & Lee, 2019) Also, and very pertinent here, is study of MSC therapy in ARDS of experimental influenza pneumonia, with aerosol application of MSC^exos^ directly into the affected lungs (Khatri, Richardson, & Meulia, 2018). This is an application of the clinical fact that applying topical therapy at the disease site offers the best therapeutic index, as is done routinely with aerosol corticosteroids applied in allergy patients with atopic allergic nasal rhinitis and asthmatic airways. Indeed, the airway exosomes that are released during influenza virus infection serve as a key component for stimulating the antiviral innate immune response (Bedford et al., 2020).

Thus, there already is a considerable literature on the beneficial use of MSC in patients with a variety of diseases. Focusing on severe acute pulmonary disease, as most pertinent here, patients with ARDS benefitted from this administration (Shah et al., 2018; Wilson et al., 2015; Zheng et al., 2014). However, note that clinical trials of ARDS with MSC^exos^ have shown greater and more consistent benefit in part due to problems of variability of viability and effects among the MSC when compared to MSC^exos^, whereas the MSC^exos^ trials have been much more consistent. Still there currently are many more worldwide clinical trials with the MSC, rather than with their seemingly superior derived MSC^exos^ (Behnke et al., 2020).

Therefore, despite the numerous growing reported data favouring replacement of MSC cell therapy with their produced MSC^exos^, some of the cognoscenti core of Mafia‐like leaders of the MSC field, still in 2020, extol and focus on and invest in treatment employing the MSC. This results in numerous current companies still gearing up to deliver to patients raw MSC from various sources. This now seems to be a distinctly uninformed and inferior approach. It ignores the greater simplicity and most importantly the far less dangerous alternative use of the MSC‐derived equally effective secreted MSC^exos^ and is despite the data favouring replacement of MSC with their produced MSC^exos^, showing that the MSC^exos^ actual mediate effects of the MSC.

### 1.9 Use of MSC and MSC^exos^ therapy in COVID‐19 pulmonary disease: Including COVID‐19 pneumonia

It is early days in the treatment of COVID‐19 patients with MSC. Recent studies from China preliminarily tested MSC treatment of COVID‐19 pneumonia (Leng et al., 2020; Liang et al., 2020; Shen et al., 2020). There was a remarkable reversal of disease and laboratory abnormalities within days of administering infusions of about 5 × 10^7^ MSC from allogeneic umbilical cord, with no toxicities. Also, there were accompanying decreases of inflammatory cytokine levels and increased anti‐inflammatory factors and suppressive IL‐10. MSC recovered from the patients showed high levels of anti‐inflammatory and trophic factors including TGF‐β. These are very encouraging results.

Recently, Brain Storm Therapeutics reported pre‐clinical study results of in house MSC^exos^ treatment for an ARDS model in mice. Intratracheal administration in the lipopolysaccharide model resulted in a statistically significant improvement in lung function, histological damage and pro‐inflammatory cytokine diminutions compared to control exosomes. Actual data have yet to be published. Finally, in a recently reported uncontrolled prospective non‐randomized open‐label cohort study of a single IV dose of proprietary bone marrow‐derived allogeneic MSC^exos^, 24 patients had a survival rate of 83%. There were significant improvements in pO_2_, neutrophil and lymphocyte counts with reduction in acute phase inflammatory and coagulopathy markers (Sengupta et al., 2020; Sengupta, Sengupta, Lazo, Hicok, & Moseley, 2020). These results and other facts about MSC, as reviewed here, have led to plans for properly controlled MSC trials in COVID‐19 patients (Atluri, Manchikanti, & Hirsch, 2020; Golchin, Seyedjafari, & Ardeshirylajimi, 2020), already begun in several countries, and as well for MSC^exos^ (O'driscoll, 2020).

### 1.10 There are a great number of advantages for the use of MSC^exos^ compared to therapy with their parental MSC

MSC^exos^ mitigate many safety concerns compared to use of viable replicating MSC. The cells may never really disappear in recipients, compared to the non‐immortal exosomes. Further in the context of COVID‐19, the know side effect that MSC IV administration may result in a coagulopathy (Riphagen, Gomez, Gonzalez‐Martinez, Wilkinson, & Theocharis, 2020) with aggregating or clumping in the viral injured microcirculation (Varga et al., 2020), perhaps due to a complement related immune reaction to the abnormal endothelium (Magro et al., 2020). This can cause lung dysfunction that might synergize with the COVID‐19 pneumonia effects. Similarly, the tendency toward similar MSC‐associated peripheral vascular micro aggregates might synergize with the vascular clots of the COVID‐19 disease process causing central or peripheral vascular insufficiency leading to heart attack with cardiac arrest, stroke, or pulmonary embolism (Klok et al., 2020; Poissy et al., 2020). In this time of hyper media coverage of COVID‐19 virus, a few such cases would dissuade thousands from considering the MSC approach. As noted MSC^exos^ have significantly less stimulatory HLA‐complex molecules and surface co‐stimulators (Saeedi et al., 2019). Thus, they are less innate immune stimulatory, nor acquired immunogenic, so that even allogeneic sources of MSC^exos^ have intrinsic tissue and cell preferential binding targeting ability to chosen target acceptor cells via their surface signature bar codes, compared to the MSC for which the peripheral tissues have no known receptivity.

MSC^exos^ are durable in culture for large scale manufacture, so that many can be harvested by repeated collections of conditioned media from the same adhering MSC donor cells in bioreactors with huge surface areas. Thus, MSC^exos^ have reduced time and cost for producing therapeutic patient material. Further, biogenesis and secretion of the MSC^exos^ can be boosted several fold by incubation in neuro bioamines (Wang, Bonacquisti, Brown, & Nguyen, 2020) .They are non‐viable but instead are subcellular particle organelles without functional genomic DNA, so they pose no risk of transferred abnormal chromosome numbers (aneuploidy), nor development of cancer promoting mutations and consequent oncogenicity.

The safety of exosomes in humans is emphasized by the fact that there have been billions in blood plasma transfusions containing trillions of exosomes over many decades in diverse patients without problems, whereas MSC as cells are difficult to standardize or predict actions of given lots in vivo in patients. On a practical level MSC^exos^ can be freeze‐thawed at least once without toxic cryo‐preservatives, with no lost activity for months to years, and it has been reported that they can be freeze‐dried (El Baradie et al., 2020), for superior storage, allowing on site administration without refrigeration. This can result in uncomplicated use on hospital wards or in poorer countries with low technology medical facilities. Thus, the MSC^exos^ are available for immediate use post thawing without washing compared to cells for easier routine hospital use. Therefore, they are an ‘off‐the‐shelf’ therapeutic with less manufacturing and storage costs.

MSC^exos^ represent a thousand‐fold purification as the actually effective component of MSC, via reduction in size and volume and have very different proteome and transcriptome compared to parental cells; obviously important considering that these in fact are responsible for the biologic effects of the MSC, that is most often mediated by the miRNAs that they deliver. As non‐cells that are non‐proliferating and not subject to differences in viability; MSC^exos^ can be more precisely standardized per dose and duration of biologic action, compared to the difficult variability of live MSC. Therefore, several reviews highly recommended that MSC^exos^ hold great potential for targeted tissue repair (Gowen, Shahjin, Chand, Odegaard, & Yelamanchili, 2020; Pashoutan Sarvar, Shamsasenjan, & Akbarzadehlaleh, 2016; Phinney & Pittenger, 2017; Yin, Wang, & Zhao, 2019; Zhao et al., 2019).

It is of course true that in this instance as in others, it is most proper to do a direct comparison of MSC to MSC^exos^, as we have done in our studies of SCI of rats (Lankford et al., 2018). This hopefully will be done in some cases of COVID‐19 treatment in research oriented units. However, the exigencies of clinical ICU use in the current COVID‐19 situation, and the arguments made here, convince the clinician in me that for beneficial effects, greater convenience in diverse medical environments and less complexity and problems, that we recommend moving directly to the MSCexos in most routine situations. The superior MSC^exos^ alternative is indeed now beginning to attract more research and commercial considerations with attempts by industry to develop new methods for generation and isolation of MSC^exos^ that will simplify patient use.

Obtaining, enriching and characterizing MSC^exos^ does need special efforts that currently are being done at some centres and by some companies and those that offer exosome isolation services. It is likely that this is being contemplated by the ATCC that already offers human bone marrow and adipose‐derived MSC. In the end, producing the MSC^exos^ is no more complex then producing the MSC, but in comparison, when available, have the advantage of an ‘off the shelf’ reagent. Certainly, use of the highest quality MSC product exosomes to avoid possible increased risk of coagulopathy (Moll et al., 2020), in matched, randomized, controlled trials should be the rule (Gorman et al., 2020).

## 2 COVID‐19 CONVALESCENT PLASMA CONTAINS IMPORTANT EXOSOMES LIKELY INFLUENCING RESULTS OF THERAPY

As noted, exosomes are nano‐sized extracellular vesicles made by all cells and are present in all body fluids. They contain greatly varying RNAs, proteins and lipid mediators that can alter the function of targeted cells nearby for paracrine transfers and in far away organs via transit of the vesicles in the blood plasma for endocrine transfers. Especially important is that exosome miRNAs transferred to targeted acceptor cells can epigenetically alter functional properties since exosomes are the chief mechanism for regulation of the behaviours of different and distant cells genetically by such RNA transfers. Exosomes can act epigenetically by their transferred miRNA inactivating cytoplasmic mRNA encoding proteins that act to alter nuclear DNA by methylation and other means to change gene expression.

Exosomes in transit in the plasma have been studied in a variety of conditions because they can be markers of diseases, severity of disease and selection of therapies. Blood plasma exosomes are especially useful in cancer for ‘liquid biopsies’ not only for diagnosis and prognosis, but also for following their properties of mediating tumour induced resistance to radiation (Ni et al., 2019) and drug therapies (Steinbichler et al., 2019). As example here in COVID‐19 ARDS patients, there can be a puzzle about the beneficial patient use and dose of corticosteroids and NSAIDs (Russell, Moss, Rigg, & Van Hemelrijck, 2020), for which examination of convalescent plasma exosomes might play a significantly helpful role. In fact, one related study in SARS patients with progressive ARDS, compared convalescent plasma therapy to 1.5 g pulsed methylprednisolone and found that the plasma gave significantly better results (Soo et al., 2004). Thus, the convalescent plasma exosomes can be an indicator of intercellular communications among the cells of the immune system to importantly serve towards choices for particular patient therapies.

### 2.1 Exosomes may have been involved but unmeasured in the favorable history of convalescent plasma therapy of prior viral diseases

Prior positive therapeutic findings concerning exosomes in convalescent plasma include severe infections with RNA viruses (Chahar, Bao, & Casola, 2015). Mostly beneficial effects that may have been due to exosomes in the employed convalescent plasma were obtained in relatively poorly controlled recent studies of viral diseases that were antecedent to COVID‐19. These included infections with related coronaviruses like SARS (Cheng et al., 2005; Yeh et al., 2005) and MERS (Arabi et al., 2016; Ko et al., 2018), as well as Ebola (Kraft et al., 2015; Mulangu et al., 2019), avian influenza (Kong & Zhou, 2006) and influenza pneumonia (Luke et al., 2010; Mcguire & Redden, 1918; Zhou, Zhong, & Guan, 2007). Generally there seemed to be an overall 60–75% improvement in severity or death rates.

Three influenza studies deserve further mention. In one, 93 patients with severe pandemic 2009 influenza A (H1N1) virus infection, in a prospective cohort study were treated with convalescent plasma having a viral neutralizing antibody (Ab) titer of ≥1:160, vs controls that were matched by age, sex and disease severity (Hung et al., 2011). Mortality in the treatment group was significantly lower than in the non‐treatment group (20% vs. 55%; *P* = 0.01). Subgroup analysis demonstrated that plasma treatment was associated with significantly lower viral load, and levels of IL‐6, IL‐10 and TNF‐α, vs. the control group (*P* < 0.05).

Another study published in 2019 examined anti‐influenza immune plasma in patients with severe influenza A in a prospective randomized, double‐blind, phase 3 trial; comparing clinical efficacy of convalescent plasma with high titer anti‐influenza A Ab to convalescent plasma with low titers (Beigel et al., 2019). There was overall benefit, but no difference was found employing high titer versus low titer Ab‐containing plasma, as also was found in the prior phase 2 trial. This raises questions about the amount or class of Ab needed or efficacious non‐Ab factors in the plasma, such as perhaps involved immune convalescent exosomes in both lots of plasma acting independently of the actions and levels of immune Ab. Further, 34% of those treated experienced serious adverse events including ARDS and allergic transfusion reactions; highlighting the usually rare but possible potential hazards of convalescent plasma.

The third study was a systematic review and meta‐analysis of the effectiveness of convalescent plasma and hyperimmune immunoglobulin for the treatment of severe acute respiratory infections of viral aetiology in several related studies published between 1918 and 1925. These showed a 75% reversal of case‐fatality rate in the treated group vs the nontreated controls (Mair‐Jenkins et al., 2015). Overall, this meta‐analysis of convalescent plasma therapy pooling data from the 1918 influenza pandemic and other viral disease studies sustained the positive conclusions of the individual past studies. Note further that in the mid‐20th century, convalescent sera and plasma were used to seemingly be able to stem outbreaks of other viral diseases such as poliomyelitis (Park, 1932), measles (Gallagher, 1935; Park, 1926) and mumps orchitis (Rambar, 1946). Additionally, there was a favourable meta‐analysis of studies using convalescent blood products to treat the pandemic Spanish influenza pneumonia of 1918–1919 (Luke, Kilbane, Jackson, & Hoffman, 2006). Therefore, there is a long history over many decades of repeated clear benefit of specific convalescent plasma in a variety of human viral diseases.

### 2.2 Unusual properties of exosomes possibly contributing to the positive effects of convalescent plasma

In contrast to convalescent Ab, convalescent exosomes act on intercellular processes and thus can inhibit viral pathogen proliferation and transmission directly (Rodrigues, Fan, Lyon, Wan, & Hu, 2018; Zhang et al., 2018). In contrast to the Ab content exemplifying humoral immunity, these convalescent plasma EV can allow the host to mount a variety of effective innate and acquired cellular immune responses against pathogens, that includes interference with activating antiviral mechanisms and transferring anti‐viral miRNAs derived from a variety of responding cells to target important acceptor effector cells (Chahar et al., 2015). Indeed, in influenza virus infection, miRNAs in the induced exosomes regulate viral replication and simultaneously had a favourable influence on the host acquired immune response (Zheng, Zhou, & Wang, 2020).

A property of exosomes in convalescent plasma that could be involved concerns the structure of their membranes that can change as a consequence of infections and immune responses (Bryniarski et al., 2013; Nazimek, Bryniarski, & Askenase, 2016). This includes alterations in the quantity of structural proteins and particularly the quantity and quality of constituent lipids influencing membrane spatial configurations (Laulagnier et al., 2004; Li, Huang, Zhang, Song, & Xiao, 2019; Li, Huang, Zhang, Song, & Xiao, 2019; Zhou et al., 2020). This can result in particularly high viscosity that enables certain subsets of these small exosome EV to withstand noxious environments like hypoxia and high acidity. This likely occurs in the pulmonary microenvironment in ARDS of severe COVID‐19 patients where cell death will generate local hypoxia (Meng, Hao, He, Li, & Zhu, 2019), acidity and released digestive enzymes that are known to be resisted by certain subpopulations of particularly hardy exosomes (Benmoussa et al., 2016). As example, there is increasing evidence that immune activation of donor cells, as undoubtedly happens in viral illness due directly to the virus and also stimulated by the resulting immune response induced by viral antigen stimulation, alters donor cell production of membrane lipids to generate exosome subpopulations with unusual features such as resistance to noxious environments that may have great relevance to their role in the beneficial effects of convalescent plasma (Bryniarski et al., 2013; Nazimek et al., 2016).

Such ‘activated’ exosome subpopulations from immune and perhaps viral activated cells acquire particular surface properties (Keryer‐Bibens et al., 2006). This can include ability to be systemically active even after resisting the acid and enzymes following oral administration (Bryniarski, Nazimek, Ptak, Kormelink, & Askenase, 2020; Wąsik, Nazimek, Nowak, Askenase, & Bryniarski, 2019). Additionally, there can be newly acquired ability to bind Ag‐specific Ab free light chains (Bryniarski et al., 2013; Bryniarski et al., 2015; Nazimek et al., 2016). This can render suppressive T cell‐derived exosomes Ag‐specific (Bryniarski et al., 2013), enabling their surface anti‐peptide Ab to bind peptide‐Ag/MHC complexes on targeted APC (Bryniarski et al., 2020; Nazimek et al., 2015), allowing transfer of specific inhibitory miRNA‐150 (Bryniarski et al., 2020; Bryniarski et al., 2015; Wąsik et al., 2019). This exosome transferred miRNA leads these targeted APC to then produce secondary suppressive surface Ag/MHC‐specific exosomes to inhibit companion effector T cells mediating immune inflammation due to inhibited production of cytokines like IFN‐γ by these effector Th1 T cells finally targeted at the immune synapse (Tsuji et al., 2002). This T cell subset is most important in pathogenicity of COVID‐19 infection‐associated cytokine storm (Mehta et al., 2020), that is relevant here since viral high Ag dose exposure induction of such immunoregulatory exosomes could similarly be present in convalescent plasma.

While inhibiting infection, reducing ARDS and suppressing deleterious aspects of the immune response such as cytokine storm, Ag/MHC surface exosomes present in convalescent plasma, particularly those from APC macrophages and DC and B cells, can also serve to augment the antiviral immune response by acting as miniature nano APC (Rodrigues, Fan, Lyon, Wan, & Hu, 2018). These can induce healing M2‐type macrophages (Lankford et al., 2018; Saha, Kodys, Adejumo, & Szabo, 2017), and promote generation of the particular subset among Th2 T cells that produce immunosuppressive IL‐10 and TGF‐β  (Mcgonagle et al., 2020). A pertinent example is that exosomes used as antigen presenting vaccines containing the S protein of the coronavirus pathogen of SARS pneumonia induce helper T cell dependent high levels of neutralizing Ab (Kuate, Cinatl, Doerr, & Überla, 2007).

In the case of seriously ill COVID‐19 patients there is an increase in innate cell lineages, with a concomitant reduction in T cells. An early elevation in cytokines is associated with worse disease outcomes. Following this, patients with moderate COVID‐19 have progressive reduction in Th1 cell IFN‐γ type‐1 antiviral macrophage activation and Th17 cell IL‐17 and IL‐22 type‐3 antifungal‐like neutrophil mediated responses. New data show that with severe disease these elevated responses are maintained but can become accompanied by an increase in Th2 cell anti‐helminth‐type effectors, including IL‐4, IL‐5, IL‐13, IgE Ab and eosinophils (Lucas et al., 2020). This final shift to Th2 responses could aid differentiation of macrophages toward M2‐type, for generating pro healing exosomes (Lankford et al., 2018), possibly important in the subsequent benefits of convalescent plasma.

Many things in the biology and medicine of viral infections can function to favour the host or the pathogen. Exosomes can be tailored for function in either direction by their producing cells depending on their received microenvironmental signals in order to alter near and far target cell activities in many ways by mediating their essential central function of delivering a cargo of specific mediators. This applies especially to delivery of miRNAs. These are capable of altering function in both positive and negative directions by affecting mRNAs encoding respectively enhancers or repressors of DNA gene expression, to modulate the infection or the immune response depending on circumstances. In particular, exosome miRNAs can promote immune resistance to viral infections in several ways; such as: inhibiting pathogen infection, viral proliferation and transmission directly, and indirectly Inhibiting infection by stimulating immune resistance responses by improving functions of macrophages, NK cells, T cells and B cells (Lucas et al., 2020).

Therefore, there are important functions that could be revealed in the study of patient plasma convalescent exosomes in COVID‐19 infection that would bear consideration in selection of particular convalescent plasmas for therapy according to their potential positive therapeutic effects on the infection and on the associated deleterious immune responses like ARDS, the cytokine storm and the coagulopathy, in addition to the current singular focus on the more restricted benefits of the contained anti‐viral Ab titers.

### 2.3 In COVID‐19 infections, what are potential beneficial functions of exosomes in treatment with convalescent plasma derived from viral and immune induced host cells

To my view, considering the effects of the exosomes in the convalescent plasma needs to be done sooner rather than later. Looking over prior knowledge of exosomes and especially their role in immune regulation, it seems that plasma during the COVID‐19 infection, especially in those patients with the cytokine storm and ARDS may initially be augmentative during this active disease phase. They likely are derived from the over blow innate immune cell response of the over stimulated macrophage family of Mac 1‐type subsets stimulated by TNF‐α, and subsequently still during active infection by the viral antigen activated Th1 cell and Th17 T cell‐derived cytokines; respectively INF‐γ and IL‐17 stimulated donor cells.

With disease progression towards resolution and beyond, biology of the exosome response likely converts to reflect their healing positive aspect also occurring in other convalescent responses. Here there would be more EV generation from healing and trophic M2‐type macrophages producing immunosuppressive IL‐10 and TGF‐β, and perhaps also from regulatory Th2 cells also making immunosuppressive exosomes (Lucas et al., 2020), and also IL‐4, IL‐13 and IL‐25. Thus, most experience and data suggest that convalescent plasma exosomes should be healing and trophic, derived from multiple cell sources, and likely transmitting miRNAs leading to epigenetic effects pertaining to such beneficial responses (Hassanpour, Rezaie, Nouri, & Panahi, 2020; Okoye et al., 2014; Yoshikawa, Teixeira, Sato, & Oliveira, 2019).

Therefore, it is postulated that activated exosomes from immune stimulated regulatory and suppressor T cells and M2‐type macrophages may make a very significant contribution to the beneficial effects of convalescent plasma or possibly account for the benefit. This would be beside and beyond the developed crucial high affinity IgG anti‐COVID 19 Ab of the acquired T cell mediated secondary B cell response. In fact, there may be Ag‐specific Ab actually on the surface of the convalescent exosomes (Bryniarski et al., 2013; Bryniarski et al., 2020; Wąsik et al., 2019), molecularly synergizing with these acquired Ag‐specific soluble immune Ab. Further, we have experimental preclinical data demonstrating suppressive synergy between the induced Ag‐specific Ab and the Ag/MHC‐specific immunoregulatory APC‐derived exosomes in this situation (Nazimek, Bryniarski and Askenase, unpublished).

Up to now there have been few observations of exosomes in the plasma of patients with the COVID‐19 infections and its syndromes. In adults, plasma blood tests early in COVID‐19 infections for studying exosome phenotype, proteome and transcriptome might be useful as ‘a liquid biopsy’ in diagnosing those patients that will go on to severe sudden pneumonia and the need for artificial ventilation, as well as predicting the likelihood and extent of the cytokine storm syndrome. Similar analysis of plasma exosomes might be useful in distinguishing the paediatric patients that will go on to have the severe Kawasaki disease‐like MIS affecting the heart and great vessels.

Per treatment, firstly COVID‐19 convalescent plasma analysis of contained anti‐viral Ab is thought useful for selecting particularly strongly therapeutic lots for treatment of the infections and/or induced associated syndromes. This is based on in vitro assay of the acquired infection‐associated viral antigen‐specific IgG Ab titers against the virus that if used for treatment early in the illness could help block the COVID‐19 viral spike protein crucial pathogenetic interaction with the specific viral receptor ACE2 on bronchial epithelial cells and/or bind virus to clear the agent via complement and IgG Fc‐dependent actions.

### 2.4 In COVID‐19 infections, how really important are the Ab of convalescent plasma in resistance to the virus?

There are few or any viral illnesses that are cleared by Ab alone; this depends more crucially on cellular immunity, such as mediated by cytotoxic CD8^pos^ T cells and NK cells, as well as cytokine producing effector Th1 T cells (Frey, Krempl, Schmitt‐GräFf, & Ehl, 2008; Rosendahl Huber, Van Beek, De Jonge, Luytjes, & Van Baarle, 2014). In fact, it could be argued in the extreme that the Ab in convalescent plasma have little to do with any beneficial effects. Consistent with this view particularly in covid‐19 infections are the following:

1. Severe COVID‐19 infection cause dysfunction of the immune system including aberrant helper T cell mediated IgG Ab responses (Lucas et al., 2020),
2. Successfully immune cleared patients have great variability in Ab levels, with unusual declines; some to base line soon after the illness (Seow et al., 2020),
3. Some patients with very little Ab response still can effectively clear COVID‐19 (Goetz, Yang, Greene, & Zhu, 2020),
4. Patients under treatment with B cell depleting anti‐CD20 monoclonal Ab were able to recover (Thornton & Harel, 2020);
5. In fact, patients with genetic Ab deficiencies are able to clear the illness (Soresina et al., 2020), and thus compared to those with Ab are less sick. Therefore, in this case Ab may favour progression of the viral illness (Quinti et al., 2020),
6. For influenza, when convalescent plasma therapy was separated into hi and lo tittered Ab levels there is no difference in eventual clinical effects (Beigel et al., 2019),
7. COVID‐19 patients have unusual depletion of lymphoid germinal centres and contained follicular helper T cells and some B cells, with unpredictable subnormal Ab responses (Kaneko, Kuo, & Boucau, 2020),
8. SARS‐CoV‐2 antibodies exhibit limited somatic hypermutation (Kreer et al., 2020),
9. Most convalescent plasma samples obtained from individuals who recover from COVID‐19 do not contain high levels of neutralizing Ab activity (Robbiani et al., 2020).
10. Recently, patients from Hong Kong and Los Vegas were reported that had documented re‐infection to COVID‐19
11. A substantial minority of COVID‐19 patients do not fully recover,
12. According to the WHO, there is not enough evidence about the effectiveness of antibody‐mediated immunity to guarantee the accuracy of an ‘immunity passport’ or ‘risk‐free certificate’; stating that individuals are therefore immune to a second infection, because they have received a positive test result and thus may ignore public health advice. The use of such certificates may therefore increase the risks of continued transmission advice. The use of such certificates may therefore increase the risks of continued transmission.
13. All the while there is a well established robust anti‐COVID‐19 viral T cell response (Sekine et al., 2020)

To our view, the use of COVID‐19 convalescent plasma for its content of acquired immune Ab must additionally consider the role, in this therapy, of the billions per millilitre of convalescent exosomes contained in the plasma. Very different than Ab, these likely mediate functions of viral activated immune modulating cells due to transferred miRNAs acting epigenetically. Therefore, these immune activated plasma convalescent exosomes may importantly be crucially involved in the positive effects of the plasma beyond the contained immune Ab, or could possibly be the sole factors inhibiting COVID‐19 pathogenesis. Mechanistically, the exosomes represent the cellular immune system upon which immune anti‐viral resistance really relies. They often act on the macrophage family (Nazimek et al., 2015) stimulated by and stimulating pathogenic T cells and most importantly on antigen presenting cells upon which activation of the truly significant effector T cells depend (Bryniarski et al., 2020; Bryniarski et al., 2015; Lankford et al., 2018; Nazimek et al., 2015; Wąsik et al., 2019). At a minimum, it would seem prudent to pre select potentially therapeutic convalescent plasma with the best Ab and the best exosomes to produce the most optimum convalescent plasma therapy for very severely affected COVID‐19 patients.

### 2.5 A preclinical model with Ag‐specific Ab coated suppressive exosomes in the convalescent immune plasma

In experiments in mice, repeated Ag high dose induced immune tolerance was designed to model and thus be similar to the immune experience of the great and multiple viral Ag load of viral infections, like with COVID‐19. We initiated these studies following clinical observations of tolerogenic suppression of the immune system as a regular part of many viral infections (Bouhdoud, Villain, Merzouki, Arella, & Couture, 2000; Ou, Zhang, Huang, & Moskophidis, 2008; Saeidi et al., 2018). In the repeated preclinical high Ag dose exposure system, there is generated suppressive CD8^pos^ T cells that act by producing suppressive exosomes that are anti‐inflammatory and anti‐immunologically suppressive (Bryniarski et al., 2013; Nazimek et al., 2016)., Moreover, these suppressive exosomes are uniquely Ag‐specific due to a coating of specific Ab‐derived free light chains from neighboring activated B cells (Bryniarski et al., 2013; Bryniarski et al., 2020), These Ag‐specific exosomes are found in body fluids, supernatant of lymph node and spleen cells, and importantly, also in the convalescent plasma (Bryniarski et al., 2013). Like the co‐incident possibly protective plasma whole Ab, these exosome‐coating Ab light chains can be quite Ag‐specific. Thus, these Ag‐specific suppressive exosomes can easily be enriched and isolated by employing their specific Ag‐binding for Ag‐affinity column chromatography (Bryniarski et al., 2013; Bryniarski et al., 2020; Wąsik et al., 2019). It is therefore possible that analysis of the function of relevant exosomes in COVID‐19 convalescent plasma, it can be shown that some of these EV may be viral Ag‐specific or Ag/MHC‐specifically suppressive of effector T cells of the illness due to the induced high vial Ag tolerogenesis.

### 2.6 Development of elite Ab to combat serious CIVID‐19 illnesses

Relating this to COVID‐19 convalescent plasma, these procedures can provide a route to determining Ab variable‐region gene sequences for development of biotechnology engineered monoclonal COVID‐19 Ag‐specific high affinity IgG Ab for more definitive treatments. These could be developed for therapy of severely affected COVID‐19 patients before emergence of effective drugs for treating the infection or most definitively specific vaccines for preventing the infection. Further, determining the convalescent plasma exosome carried and transferred miRNAs of the crucial suppressor exosomes that is responsible for healing, via RNA sequence cloning may isolate crucial inhibitory miRNAs useful themselves in treating the patients. This convalescent exosome‐associated miRNA may lead to the cytosolic targeted mRNA of acceptor cells that they affect and subsequently on to the upstream involved DNA genetic sequence for possible development of molecular therapy for COVID‐19 diseases as well. The currently available exquisitely sophisticated molecular methodologies may mean that these steps can be accomplished in a very short time, compared to the necessary much longer time to develop a safe, useful and effective vaccine or appropriate drug therapy.

Finally, in planning convalescent plasma therapy of COVID‐19 patients, there will be a focus on determining the plasma samples with the most effective Ab and titers using pre‐testing with assays like in vitro viral neutralization. The argument here is that additional determination of the beneficial biologic effects of contained convalescent exosomes in the plasma should also be performed for constructing more optimal total protective biologic therapy. This would be from more precise selection of the most optimal plasma samples to use for the most effective treatment of very sick COVID‐19 patients. Also, for some investigators there will be a natural tendency to isolate the Ab in the convalescent plasma to express potential therapeutic titers in μg/ml. There additionally will be refinements using sophisticated technologies aimed at isolating especially effective elite sub populations of the most effective broadly neutralizing ab, as seemingly achieved for HIV (Mendoza et al., 2018), and the immunoglobulin variable portion genes of the very few cells that produce them. A related approach is to attempt capturing and recreating diverse antibody repertoires as a multivalent recombinant polyclonal anti‐COVID‐19 Ab drug (Keating et al., 2020).

### 2.7 A potentially relevant cautionary note: Exosomes in convalescent plasma could mediate negative therapeutic effects; overall opposing the proposed beneficial effects of Ab

Although there is much data suggesting that the exosome component of convalescent plasma likely contributes to the positive effects, like all things in biology there can be negative properties in some instances. These may be determined to be detrimental, so that removing exosomes from the convalescent plasma may result in a better therapeutic agent for COVID‐19 patients. Exosomes definitely can go in both directions dependent on the phase of illness and other aspects. At the height of the cytokine storm and ARDS there is good reason to think that plasma exosomes mediate pro‐disease effects. In convalescent plasma from later in the process, exosomes likely will have evolved properties to favour suppression of disease as reviewed here and thus be a useful aspect of convalescent plasma. However, the opposite may be true for some of the exosomes and thus to proceed most effectively it will be needed to isolate and then then remove the exosomes from the Ab in the convalescent plasma. Use of serum instead of plasma does not help since it contains most of the exosomes found in the plasma.

There is a paucity of knowledge about the clinical effects of exosomes in convalescent plasma per se, and no data are yet available about this aspect of COVID‐19 infections. There definitely can be negative effects of exosomes induced during the height of the infection as viruses generally use host production of exosomes for their benefit. Thus, an important aspect is that viral infection stimulation of host cell exosome production favourable to thwarting the host is a central mechanism of how the virus controls the host. This might carry over into exosomes in the convalescent plasma and thus call for removal of the exosomes to improve the potential positive effects mediated by the acquired immune system production of beneficial convalescent Ab.

Further, like in suppression of the host effector T cell response in cancers we postulate that exosomes with negative actions on the effector immune system in some of the potential COVID‐19 therapeutic plasmas may express check point inhibitory surface PD‐L1 (Chen et al., 2018; Poggio et al., 2019), or other co‐inhibitory receptors, as found in parasite infections (Dookie et al., 2020). These would be induced by the virus to thwart the host effector T cell mediated anti‐COVID‐19 responses, and will have to be removed to obtain the best effect in treatment of the ARDS and cytokine storm processes. Finally, note conditions could emulate the situation in EBOLA infections, where induced host exosomes can express a viral encoded Ag called VP40 that is a suppressor of effector T cells (Pleet, Demarino, Lepene, Aman, & Kashanchi, 2017), and similarly would have to be removed.

### 2.8 Current treatment studies of convalescent plasma for patients with severe COVID‐19 syndromes

It will be at the least several months or more until really effective drug treatment for active infection will be available to replace convalescent plasma. Additionally, an even longer time must elapse until effective vaccines are available to specifically prevent infection. Therefore, for now and many months ahead, given the large continuous number of new patients and the substantial severity and death rate, use of convalescent plasma supposedly for its Ab content, will be the only viral‐specific treatment available. Appropriately, there are developing individual and inter connected consortium programs with active participation of professional statisticians and experts on designing controls, for organizing and directing important large scale trials. The most prominent is a USA FDA investigational convalescent plasma transfusion national Expanded Access Program (EAP) for COVID‐19 led by Dr. Michael Joyner of the Mayo Clinic. It involves 40 leading US medical centres, nearly 3000 acute care sites in the US, as well as 12,000 physicians and 72,000 patients.

Initially, this group performed a meta‐analysis to determine the effect of COVID‐19 convalescent plasma on mortality and aggregated patient outcome data from a group of prior trials, including: randomized clinical trials, matched control trials and case‐series studies (Joyner, Klassen, Senefeld et al., 2020). Fixed‐effects analyses demonstrated that hospitalized COVID‐19 patients transfused with convalescent plasma exhibited about a 57% reduction in total mortality rate compared to matched‐patients receiving standard treatments (25%, *P* < 0.001) for a highly significant 32% net improvement (Joyner, Bruno, Klassen et al., 2020). These historical data provide evidence favouring the efficacy of human convalescent plasma as a therapeutic agent in hospitalized COVID‐19 patients and stimulated embarking on the US FDA investigational convalescent plasma transfusion national Expanded Access Program (EAP) for COVID‐19.

It had been hoped that their resulting convalescent plasma treatment studies would be aimed at reaching scientifically guided conclusions pertaining to possible routine use of convalescent plasma for treating hospitalized active cases of COVID‐19 infections with severe complications that would include evaluation of the contained convalescent exososmes. However, in the current rush to find effective treatments for severe COVID‐19 patients, the studies that are on the way using convalescent plasma treatment are entirely focused is on the contained immune Ab; with no planned consideration of the important and perhaps crucial convalescent exosome aspect. Unfortunately, the FDA views convalescent plasma as a single entity with effects solely due to the immune Ab component.

Preliminary publications from this group have been posted as not reviewed preprints; the first confirming the safety of convalescent plasma in 20,000 patients, as expected (Joyner, Bruno, Klassen et al., 2020). Unfortunately, the first study of effectiveness was inadequate. It involved a large number of 35,322 transfused patients with heterogeneous demographic and clinical characteristics. Unfortunately, there was no randomization, no placebo controls, nor better yet blinded placebo controls (Joyner, Senefeld, Klassen et al., 2020). Further, the sole end point was survival after beginning early in the illness, without solid criteria defining as to what early exactly was by objective clinical criteria, for the obvious need of homogeneity of the data. The results showed that the death rate after 7 days was 8.7% in patients treated within 3 days of starting, with a greater death rate at 11.9% in those transfused 4 or more days after diagnosis (*P* < 0.001). This meager 3.2% difference over only an interval of 4 days was statistically significant because of the huge number of patients involved, but clinically it is not considered very important.

Further, in this study there was a just significant relationship between Ab titer and efficacy. Patients receiving high IgG Ab containing plasma had 7 day mortality of 8.9%, but with medium IgG titers it was worse at 11.6% and with low tittered Ab it was even worse at 13.7%. These small differences among such high numbers of patients, showing that with high Ab vs. medium Ab tittered plasma there was only a 2.5% difference, and for medium Ab versus low titer Ab it was just 2.1%, were barely significant at *P* = 0.048. Again, considering the large size of the group, a substantial question is raised by these meager positive data that all is not as was presumed, i.e., convalescent plasma does not seem to be very effective. Further unfortunately, sera were tested with a binding Ab qualitative assay employing a commercial kit, and not a quantitative specific anti‐COVID‐19 infective assay, that was said to correlate.

### 2.9 The FDA issues an Emergency Use Authorization (EUA) for convalescent plasma as potential promising COVID‐19 treatment, on 23 August 2020

This authorization was despite the lack of proven efficacy, and the negative opinions of Drs, Anthony Fauci, Head of the Allergy and Infectious Diseases Institute of the NIH and Francis Collins, the overall head of the NIH. The main basis for this decision was said to be the FDA sponsored Mayo Clinic non‐peer reviewed, preprint posted study described above. As noted, the authors concluded in this 35,000 patient study that the benefit shown between early days 1–3 compared to day 4 showed that the treatment works. This was a highly debatable conclusion. There was no table comparing early and late treated patients, no multivariable analysis, no fixed effects model, no adjusting for time; nothing except a single stratified table. Since most of these really matter to outcomes, these are very basic limitations.

Thus, with a panic prescription response to COVID‐19 driven by President Trump's personal re‐election urging, resulting in the politically driven FDA emergency authorization, we may not come to know if convalescent plasma is effective against COVID‐19. At a press conference, The President said it was ‘proven to reduce mortality by 35 percent’; even though the study did not have controls and even worse, the 35% number was about a highly selected subgroup of patients and a massive mis‐communication. It was falsely stated that out of every 100 patients 37 will be saved; a gross misunderstanding of a relative risk of 0.63 communication. It was falsely stated that out of every 100 patients 37 will be saved; a gross misunderstanding of a relative risk of 0.63. Instead, the overall data show that out of 100 patients, three would be saved at 7 days, or 5 at 30 days (Gharbharan et al., 2020). In actually, the 37% figure was quite artificial as it was based on an extreme subgroup pulled out of the data in retrospect. The actual data were that 37% would be saved out of those less 80 years old, that were not on a ventilator and who were treated with plasma having high tittered Ab, *and* within 3 days, for a *P* = 0.03. Therefore, this was a subgroup of a subgroup of a subgroup, of a subgroup, i.e. quite cherry picked!

In fact, although safe, no one actually is sure whether convalescent plasma really works or how it works. Further, no one knows how much plasma to give, what constitutes a good donor, when is the best time in the disease course to transfuse, how much to give, repeatedly or not, who are the best recipients, whether it should be given with other therapies; and very importantly, there is no thinking about the billions of immune convalescent exosomes also administered with the supposedly effective Ab. The rush should be trying to figure out if convalescent plasma really works, or possibly what could be the cause of the lack of expected more positive effects, but the President has made that impossible since it is now likely that no trials will get funded nor enrolled.

Instead, there likely will be vicious infighting as doctors struggle to obtain the short supply of convalescent plasma for their patients, since the president has essentially announced it as a cure. Convalescent plasma will become the treatment of choice for the rich, who will besiege the short supply of doctors and plasma, for an emergency medicine that likely will not be paid for by insurers, so the rich will likely be first in line.

### 2.10 An alternate hypothesis: the hardly significant data suggest that in this instance the exosomes may antagonize the meager Ab effects

This very slightly positive, much over ballyhooed result by The President, compared to previous studies carried out in an analogous manner and the meta‐analysis of such prior studies in seriously ill patients with other viral diseases, instead had decreased mortality differences of 25–75%; a big difference (Arabi et al., 2016; Beigel et al., 2019; Cheng et al., 2005; Gallagher, 1935; Hung et al., 2011; Ko et al., 2018; Kong & Zhou, 2006; Kraft et al., 2015; Luke et al., 2010; Luke et al., 2006; Mair‐Jenkins et al., 2015; Mcguire & Redden, 1918; Mulangu et al., 2019; Park, 1932; Park, 1926; Rambar, 1946; Yeh et al., 2005; Zhou et al., 2007). Further, retrospective compiled data from more than 800 participants in a dozen prior COVID19 convalescent plasma studies found that the treatment decreased mortality from 26% to 13% (Joyner, Klassen, Senefeld, et al., 2020). These differences between results of the current huge FDA Mayo clinic study and prior experiences, suggest that something is very different in this case of use of convalescent plasma in very ill COVID‐19 patients. The result could have to do with the particularly meager role of Ab and associated other dysfunctional aspects of the particular immune response in COVID‐19 patients (Frey et al., 2008; Goetz et al., 2020; Hassanpour et al., 2020; Kaneko et al., 2020; Kreer et al., 2020; Lucas et al., 2020; Okoye et al., 2014; Quinti et al., 2020; Robbiani et al., 2020; Rosendahl Huber et al., 2014; Seow et al., 2020; Soresina et al., 2020; Thornton & Harel, 2020; Yoshikawa et al., 2019), or perhaps negative acting exosomes in the convalescent plasma.

Besides the Mayo Clinic study, for further support of its position the FDA cited two randomized clinical control trials. One in China (Li et al., 2020) and one in The Netherlands (Gharbharan et al., 2020), but none in the USA; a significant waving of the usual rules and further dubious reasoning. The randomized study from China involved 103 patients with severe or life‐threatening COVID‐19 and showed there was an anti‐viral effect, but there was no significant survival difference except in a culled subgroup, and thus the trial was ended early (Li, Zhang, et al., 2020). The Netherlands trial was a case control study also halted prematurely to reset the study after 86 patients were enrolled, that again found there was no therapeutic difference versus matched controls not receiving convalescent plasma (Gharbharan et al., 2020). Curiously, they reported the same neutralizing Ab levels in the patients as in the treatment plasm , but they did not present important control possibly negative serological data from a disease group that occurred years before COVID‐19.

Finally to pile on further per doubts about the efficacy of COVID‐19 convalescent plasma therapy is a very recent large outstanding study from India. It was conducted by the Indian Council of Medical Research, the apex medical research body in India; claimed to be the first randomized controlled trial (RCT) for plasma in COVID‐19 patients to be completed in the world, and also not yet reviewed. This was a large many many author open‐label, parallel‐arm, phase II, multicentre, RCT in thirty‐nine public and private hospitals across India of hospitalized, moderately ill confirmed COVID‐19 patients with low pO2 on supplemental oxygen but not critically ill with organ damage, randomized to either best standard care vs. two doses of 200 mL convalescent plasma transfused 24 hours apart. Over three months in early 2020 participants were enrolled respectively into 235 control and 229 intervention groups showing improvement in 18.7% of the intervention arm and 17.9% controls and equivalent mortality; a definitively negative result in this well controlled study (Agarwal, Anup, et al., 2020). However, Ab titers OF 1:80 were acceptable, there was improved symptoms and of oxygenation and faster viral clearance in patients in the intervention arm compared with the control arm, but lack of severity of patients may have blunted determination of more significant beneficial effects.

All together, these are yet other instances where the anti‐viral Ab levels in the convalescent plasma did not produce the expected result judging from convalescent plasma use in a variety of other instances of therapy in many patients with viral diseases prior to COVID‐19. These seemingly aberrant COVID‐19 data, but not at all uniquely negative results, contributes to our argument that the complete composition of the plasma; including the potentially negative acting exosomes must be determined. These striking differences in current studies showing no or quite weak effects of convalescent plasma in COVID‐19 patients compared to prior very promising data in other viral diseases, begs for considering the role of the contained convalescent exosomes, that conceivably could be found to be inhibitory, in attempting to unravel the best use of convalescent plasma.

One suitable control study from the USA that in fact was positive, was overlooked by the FDA. It was from the heavily affected Mt Sinai Hospital in New York City, with many available treated patients and disease controls. It involved 39 patients, who were compared to carefully matched severe active disease controls, and showed the value of convalescent plasma treatment. Namely, 12.8% of recipients of convalescent plasma died compared to double that number at 24.4% for the matched untreated controls, with further respectively, 72% treated versus 67% control patients discharged alive (Liu, Lin, Baine, et al., 2020). Together this and the other contemporaneous less extensive studies (Duan et al., 2020; Shen et al., 2020; Ye et al., 2020; Zeng et al., 2020; Zeng et al., 2020), showed encouraging evidence for the value of convalescent COVID‐19 plasma treatment, but left open the important question concerning the role of Ab compared to the contained convalescent exosomes.

In additional support, the FDA cited prospective trials in which control patients were not transfused due to plasma unavailability and also not‐truly‐randomized pre‐print reports showing encouraging signs of effectiveness (Abolghasemi et al., 2020; Rasheed et al., 2020; Zeng et al., 2020), as well as retrospective matched cohort studies (Hegerova et al., 2020; Liu et al., 2020; Perotti et al., 2020; Salazar, Christensen et al., 2020), and mere case series (Hartman, Hess, & Connor, 2020; Martinez‐Resendez et al., 2020; Salazar, Perez et al., 2020). We anxiously await for the results of studies in the UK, conducted by the NHS Plasma Program across its 23 main blood centres, which hopefully will provide more definitive data of well controlled studies to guide recommendations on the general role that convalescent plasma should have in the treatment of COVID‐19 patients. Also awaited are many on going proper RCTs prospective, single‐centre, phase 2 RCT that wonderfully is blinded to participants and the clinical outcome assessor, and is headed by Christina M. Eckhardt of Columbia University Irving Medical Center in New York, USA (Eckhardt et al., 2020).

## 3 CONCLUSIONS PER CONVALESCENT PLASMA

Therefore, it is a major oversight that no current or planned studies include evaluating the role of the contained convalescent exosomes in immune plasma therapy for COVID‐19. Certainly, this should be done compared to heavy investment in the much ballyhooed anti‐viral drug remdesivir that acts on viral metabolism in which the major favourable result was just that hospitalizations were 4 days shorter. At the moment, it is not clear that convalescent plasma works at all in COVID‐19 despite the mistaken FDA blessing as an approved emergency therapy that has resulted in huge recruiting programs throughout the country for a therapy that likely will have little effect. There is sufficient alternate evidence, like recovery of patients with little or no antibodies (Goetz et al., 2020; Seow et al., 2020; Soresina et al., 2020; Thornton & Harel, 2020), to postulate that the antibodies actually have little effect and that immune resistance is principally due to T cell mechanisms not represented by the exosomes that may still be in a pro‐illness mode. This would account for the observations that COVID‐19 convalescent plasma has weak beneficial effects compared to what was expected. This may be due to a dysfunctional immune response to the infection and resulting weak Ab that in the convalescent plasma may be impaired further by antagonistic exosomes. Surely in such an enormous and important effort there is room for and great need for our strong recommendation that a major effort be mounted to determine the role of and relative strengths of contained Ab versus carried exosomes; the latter potentially delivering important epigenetic function altered by contained miRNAs that might in fact may be antagonizing the meager beneficial effects of contained Ab.

## 4 OVERALL SUMMARY

There was a recent editorial by Dr. Sunny Dzik of the Massachusetts General Hospital who is the editor of *Transfusion Medical Review*, entitled: ‘COVID‐19 Convalescent Plasma: Now Is the Time for Better Science’ (Dzik, 2020). I agree completely with his arguments that after almost ten instances of use of convalescent plasma in epidemics and pandemics over more than a century we are now using convalescent plasma again, requiring at long last thoroughly conceived and controlled studies to truly establish efficacy. To this, I am also bringing attention to the newly recognized fact that convalescent plasma undoubtedly contains very biologically active exosomes mediating important aspects of the effects of this plasma therapy, and that so far are to be completely ignored in currently planned claimed better scientifically conceived studies.

Regarding MSC versus MSC, it is also committing significant medial research oversight to use MSC instead of the their produced MSC^exos^ that actually are responsible for their beneficial actions. To me, it also can be seen similarly as extreme medical research oversight to perform studies of convalescent plasma treatment aimed entirely at the Ab content, avoiding the contained exosome extracellular vesicles; i.e. the ‘Elephant in the Room’. These companion immune and viral activated exosomes in billions per millilitre, that undoubtedly play an important role in the effects of the plasma, may be crucial to the outcome of convalescent plasma treatment of very sick patients with COVID‐19 syndromes. *An important question remains: when will an investigators or administrators have the nerve (really modern scientific intelligence) to conduct a COVID‐19 preliminary study comparing the effects convalescent plasma to exosome‐depleted plasma and to the isolated exosomes?*
